# Are We Resecting Too Much Colon in Perforated Diverticulitis?

**DOI:** 10.7759/cureus.68473

**Published:** 2024-09-02

**Authors:** Vijay Naraynsingh, Miranda Maharaj, Fidel S Rampersad, Samara C Hassranah, Sandeep Maharajh

**Affiliations:** 1 Clinical Surgical Sciences, The University of the West Indies, St. Augustine, TTO; 2 Surgery, Medical Associates Hospital, St. Joseph, TTO; 3 Surgery, Regional Health Authority, Sangre Grande, TTO; 4 Radiology, The University of the West Indies, St. Augustine, TTO; 5 Surgery, The University of the West Indies, St. Augustine, TTO

**Keywords:** colon resection, diverting colostomy, laparotomy, perforated diverticular disease, hartmann’s procedure

## Abstract

Initially, the Hartmann’s procedure was done to reduce mortality in surgery cases of malignant rectal lesions, and not benign disease. However, the procedure was popularized in the management of perforated diverticular disease (PDD) in the 1970s. Herein, we present a case of a patient who had laparotomy and colostomy for PDD. During the post-operative planning for reversal of the diverting colostomy, a contrast study was done that revealed that most of the sigmoid colon was in fact healthy. In this patient, the colon was severed at the point of the perforation and exteriorized, which allowed time for the resolution of the gut inflammatory changes. Thus, Hartmann’s operation would have led to the unnecessary resection of the healthy sigmoid colon and possibly condemned the patient to an irreversible stoma. In severe PDD, where a Hartmann’s procedure is considered, one could sever the colon at the site of perforation and bring out a colostomy while tacking the closed, unresected distal end near the ostomy. Further contrast studies of the colon could assist in planning resection and anastomosis.

## Introduction

Diverticular disease is becoming an increasingly common problem, particularly in western countries including the Caribbean. According to Wong et al., patients over 45 years of age have a 33% risk of developing diverticular disease that increases substantially with age to 66% in those over 85 years of age; 10%-25% of diverticular patients develop episodes of acute diverticulitis [1]. Uncomplicated diverticulitis settles on conservative medical management in the majority of cases. However, complicated diverticulitis showing free perforation with fecal or purulent peritonitis has a mortality rate of 6%-35%; this is a surgical emergency requiring resuscitation and immediate operation [1].

Surgical management is usually a two-staged procedure, most commonly a Hartmann’s procedure with resection of the diseased colonic segment and end colostomy. In 2000, the American Society of Colon and Rectal Surgeons (ASCRS) advocated the Hartmann’s procedure as the optimal management for perforated diverticular disease (PDD) to achieve a good source of sepsis control and better restoration of bowel continuity [2]. However, the reversal of Hartmann’s is difficult and often condemns the patient to a permanent colostomy. In addition, the mortality rate of the Hartmann’s procedure ranges from 2.6% to 38.6% [1]. For these reasons, the ASCRS advised in 2014 that the Hartmann’s procedure was no longer the treatment of choice due to its complications [2]. Despite this, the procedure is still done in over 90% cases of PDD when the inflamed bowel is encountered in Hinchey 3 and 4 [3]. Most Hartmann’s operations are still not reversed due to the complicated nature of the operation, resulting in low reversal rates and poor outcomes in many patients [3]. In comparative studies, the Hartmann’s procedure had more severe complications (20%) and a reduced reversal rate (57%) versus ileostomy with lower reversal complications (0%) and higher reversal rates (90%) [2].

Herein, we present a case of PDD where, during post-operative planning for reversal of the diverting colostomy, it was found that most of the sigmoid colon was in fact quite healthy and limited resection was possible.

## Case presentation

**Figure 1:**
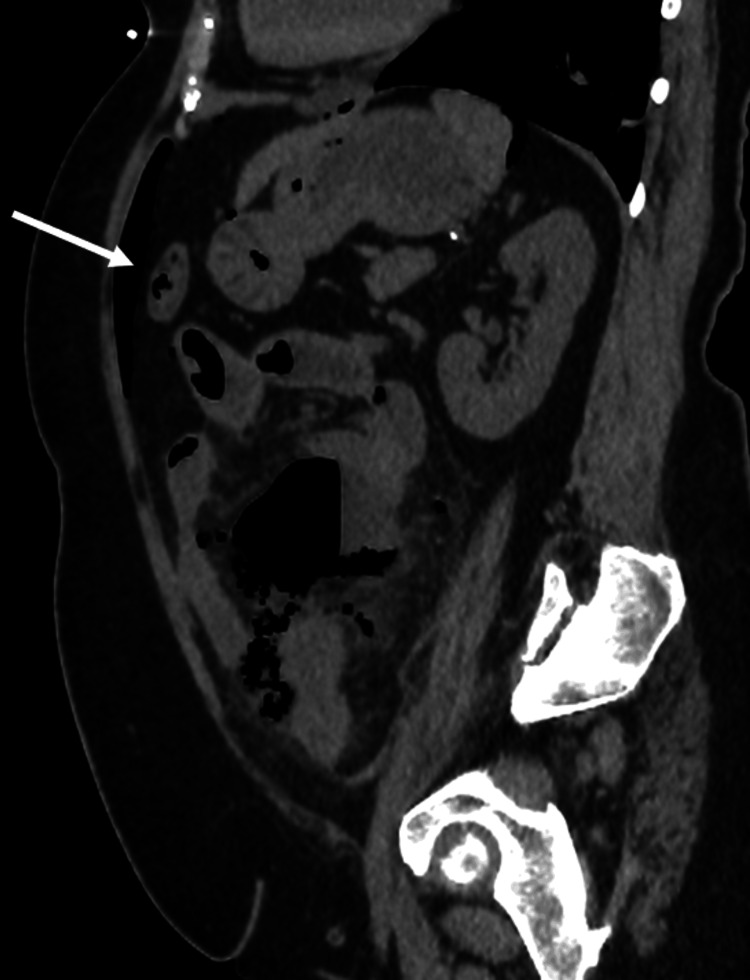
Free intraperitoneal air (arrow)

**Figure 2:**
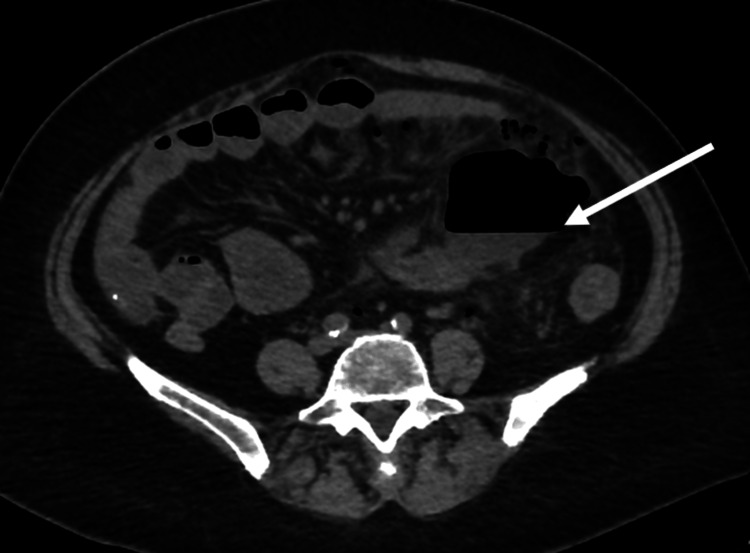
A collection with air fluid level (arrow) in the left iliac fossa

**Figure 3:**
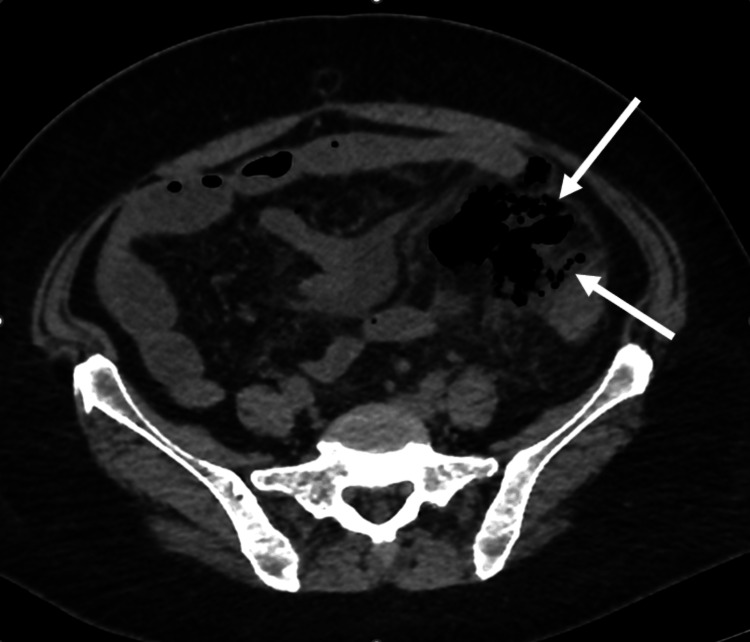
Multiple pockets of extraluminal air (arrows) in the left iliac fossa

A 65-year-old female presented with a four-day history of worsening pain especially across the lower abdomen. She was suffering from vomiting and constipation for the last two days, but there was no fever, diarrhea or hematochezia. On inspection, she looked well. She was afebrile, with pulse at 88/min, respiratory rate at 20/min, blood pressure at 120/80 mm Hg. On abdominal examination, she was tender across the lower abdomen, worse in the left iliac fossa and supra pubic area where there was marked rebound tenderness. For radiological investigation, she underwent CT that showed free intraperitoneal air, a collection (air fluid level) in the left iliac fossa and multiple pockets of air adjacent to the sigmoid colon (Figures 1-3).

**Figure 4:**
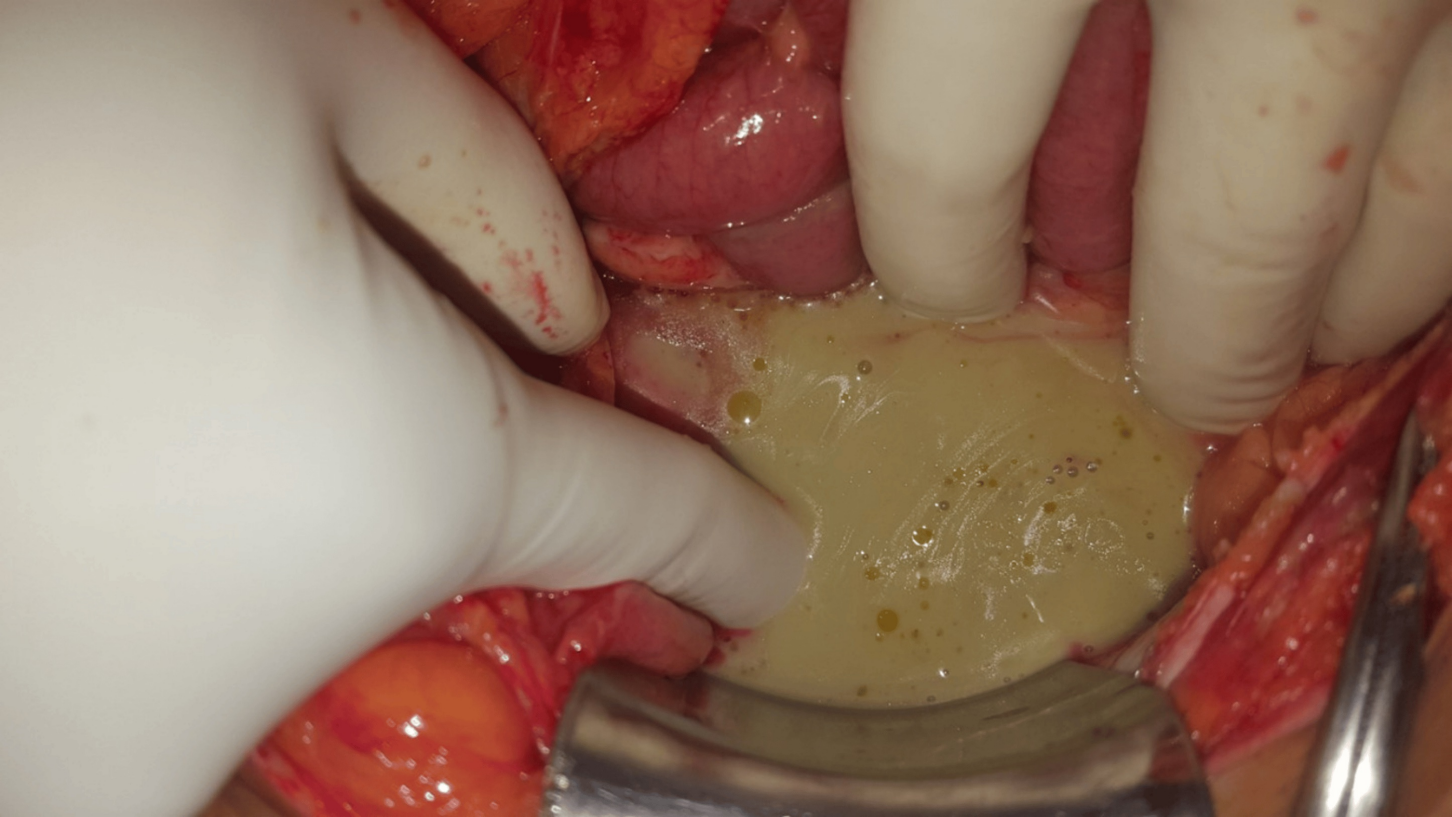
Extensive feculent contamination in the peritoneal cavity

**Figure 5:**
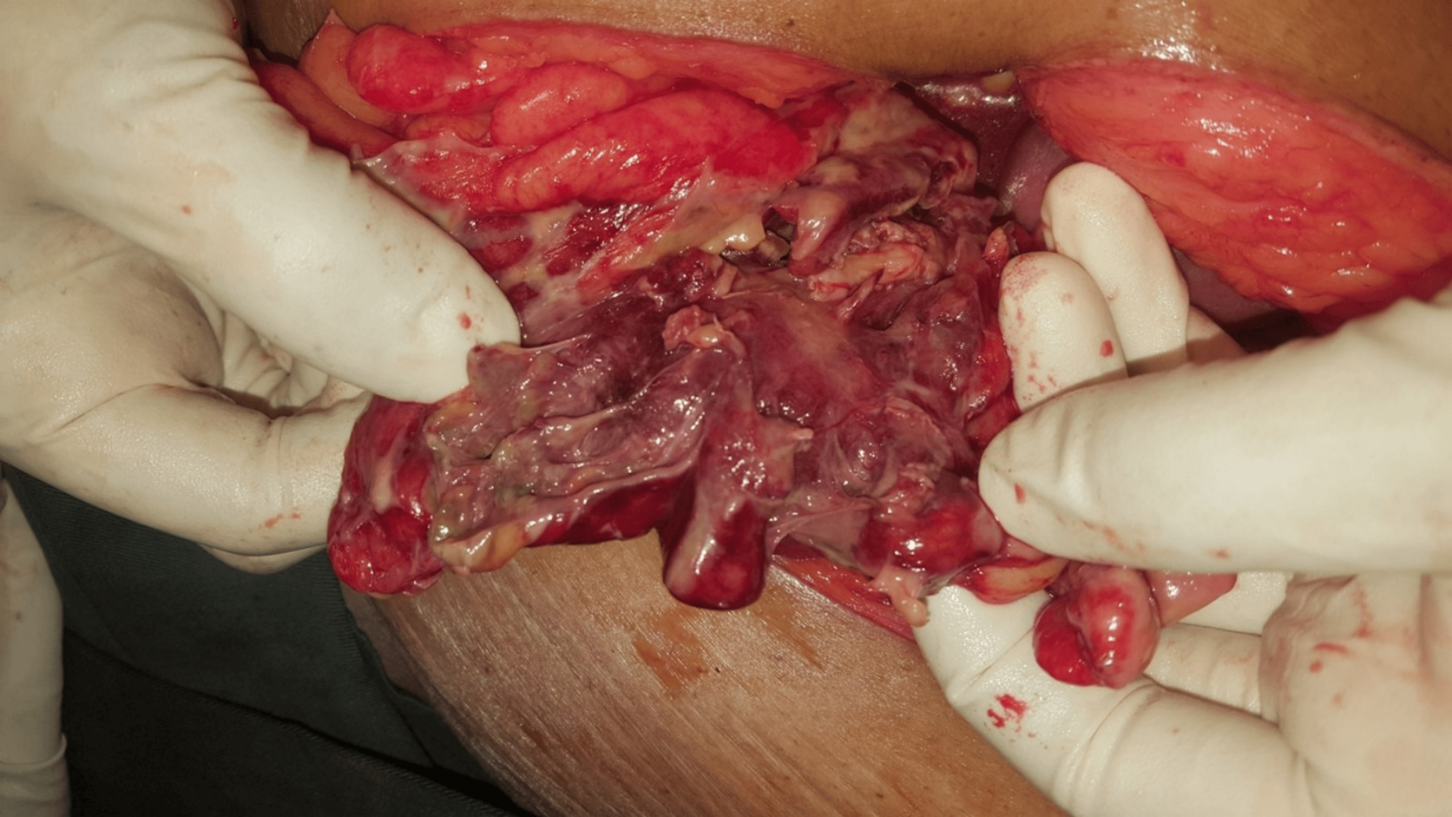
Inflamed, edematous, tattered sigmoid colon

At laparotomy, there was feculent peritonitis with large collections adjacent to the sigmoid (Figure 4). The entire sigmoid was inflamed, discoloured, oedematous and tattered (Figure 5). Only 4 cm of the highly diseased bowel was resected with the perforation. The proximal end was brought out as a colostomy. The distal closed stump was tacked to the lateral abdominal wall close to the ostomy site. The patient recovered well but developed a mild infection of the midline incision. She was discharged eight days post-operatively and had outpatient dressings for six more days until well healed. Histology confirmed diverticulitis, and no cancer.

**Figure 6:**
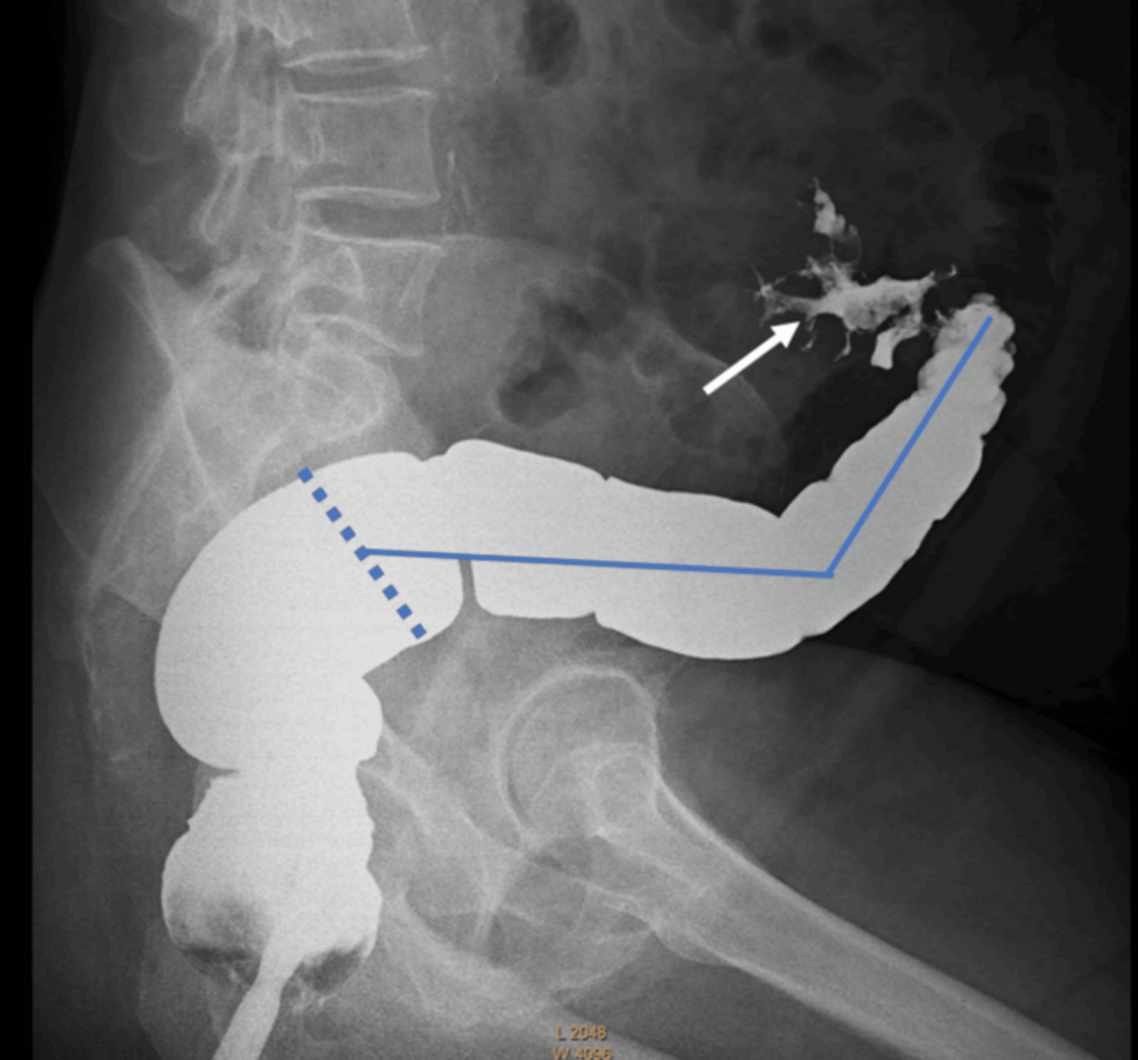
Contrast study showing 26.38 cm of the healthy sigmoid (solid line) from the recto-sigmoid junction (dotted line) to 5 cm of the highly diseased sigmoid (arrow)

**Figure 7:**
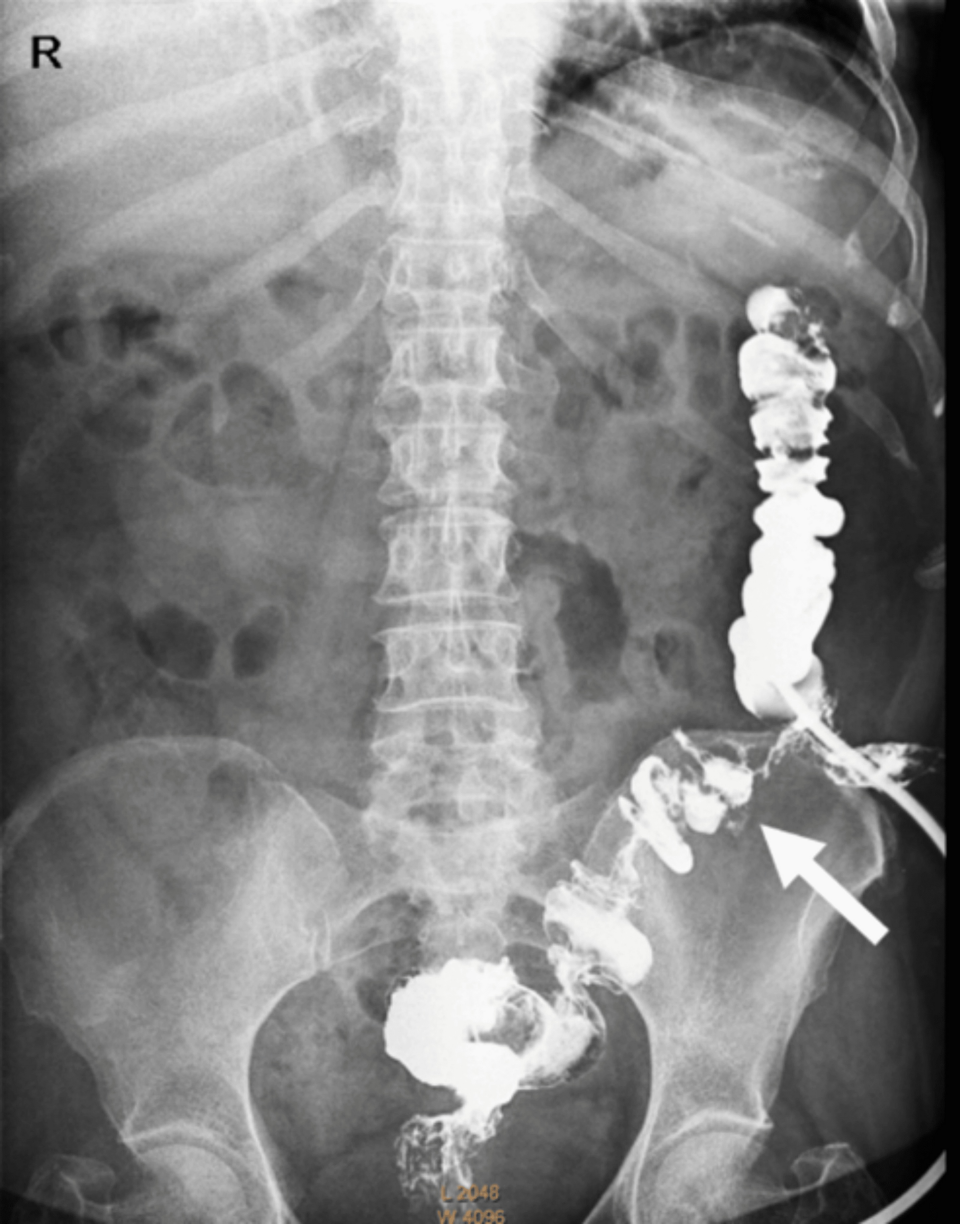
Rectal and colostomy contrast showing the distal stump (arrow) very close to the ostomy site

**Figure 8:**
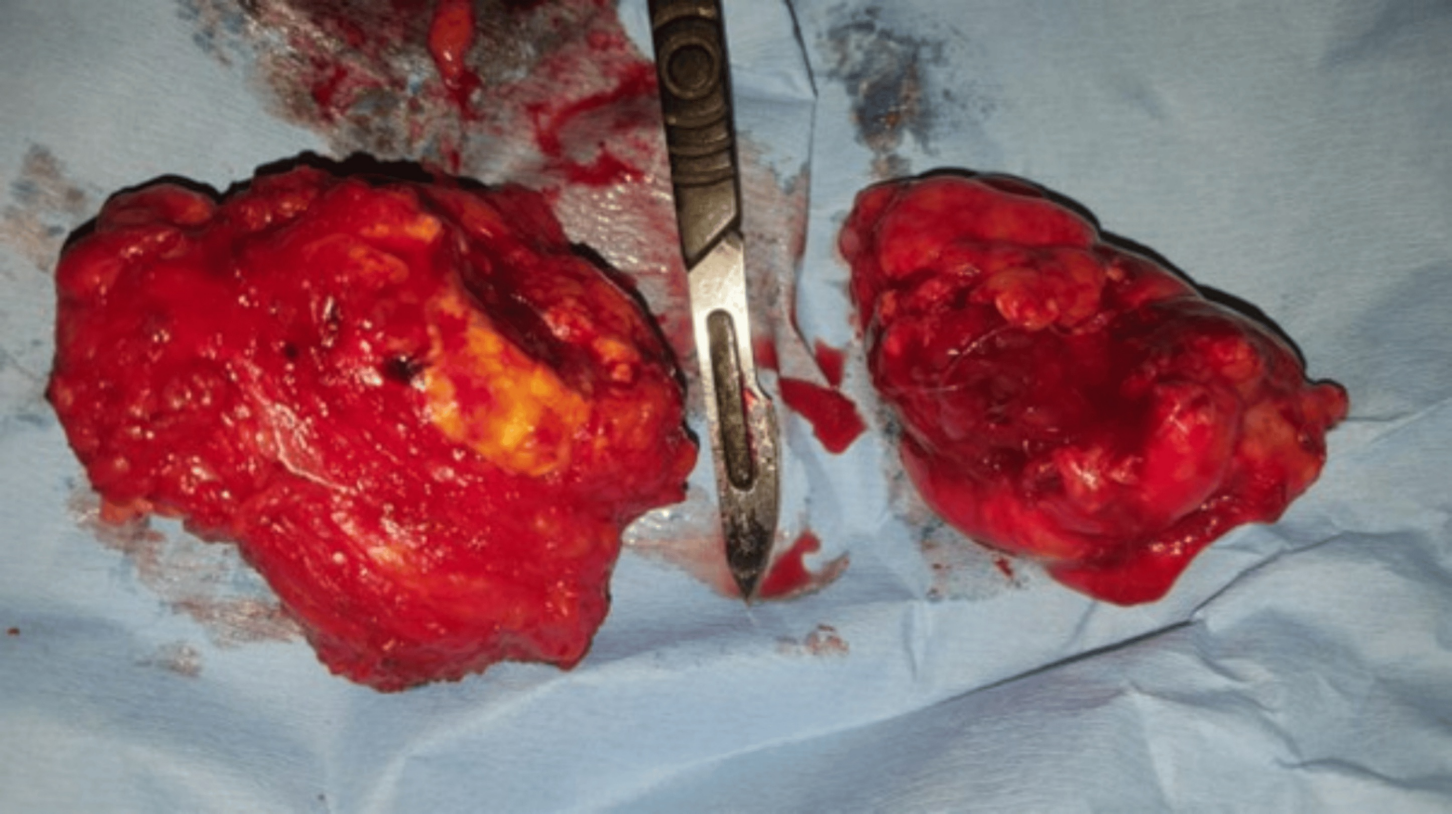
Post-operative specimens showing very limited colon resection, proximally and distally

**Figure 9:**
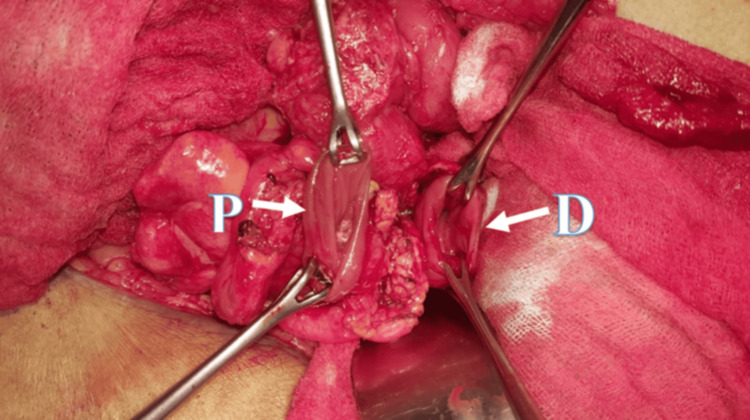
Intraoperative image of the healthy colon, at both ends proximally (P) and distally (D), prior to anastomosis

Four weeks following operation, a contrast study was done to assess the bowel with a plan for reversal. The imaging showed that most of the sigmoid colon, 26.38 cm from the recto-sigmoid junction, was in fact healthy, with only a 5-cm segment needing resection near the closed stump (Figure 6). Also, dye introduced proximally showed a healthy colon (Figure 7). At laparotomy, only a limited 6-cm resection proximally and distally revealed soft, healthy ends for anastomosis (Figures 8, 9).

No defunctioning stoma was done. She recovered uneventfully, and was discharged five days later. On follow-up, two years later, she had normal bowel actions, with no recurrent symptoms.

## Discussion

In 1921, Henri Hartmann developed the Hartmann's procedure for patients with rectal cancer as an alternative to Miles’ abdomino-perineal resection, which at the time had a very high mortality rate [4]. Hartmann’s original intention was purely to reduce mortality in surgery for malignant rectal lesions, and not for benign disease, but its popularity for PDD grew from the 1970s and was formally sanctioned by the ASCRS in 2000 [2]. They stated that for Hinchey 3 and 4 PDD, the Hartmann’s procedure is the recommended management [2]. There was then a push by the ASCRS to make the management more case specific from 2006 to 2014 [2,5]. However, by 2020, they abandoned these recommendations stating that these patients should preferably be managed by primary resection and anastomosis [6]. Despite this, in 2018, Hallam et al. reported that between 2008 and 2014, the reversal rate was only 47%, with most patients with a Hartmann’s operation condemned to a permanent stoma for benign disease [7]. Similarly, Cauley et al. revealed that out of 124,198 patients with perforated diverticulitis who underwent urgent surgical management, approximately half (67,721) had a diverting stoma. Furthermore, the majority of patients (96%) with a diverting stoma had a Hartmann’s operation and only 3.9% had a primary anastomosis with a covering ileostomy [3].

While Hartmann’s operation seems like a relatively simple procedure in the context of an acute abdomen, its reversal certainly is not. A Hartmann’s operation requires resection of the sigmoid and closure of the upper rectum as the distal stump while the proximal end of the colon is brought out as a colostomy. The removal of the entire sigmoid colon is what ensures such a low reversal rate. Studies from the United States, Australia and Canada report a Hartmann’s operation rate of 92%, 72% and 64%, respectively, for PDD [8-10]. The distance between the ostomy and the stump makes the reversal technically difficult. Even when a primary anastomosis is performed, the distal transection point is usually made on the proximal rectum, including mostly healthy colon in the resection [11]. However, a sigmoid perforation is usually only a single 3- to 10-mm defect in the affected bowel and a complete removal of the sigmoid colon, 25-40 cm, seems an extreme operation for a lesion less than 1 cm. At the time of the operation, because of the inflammatory process, the peritoneal and mesenteric edema is likely to be exaggerated. While diverticulitis may cause the gut to become scarred, thickened or strictured, in many cases the gut at the point of the perforation is normal and a primary repair can be done [12]. When the peritonitis is severe and primary resection with anastomosis is unsafe, we recommend that a Hartmann’s operation still not be performed; instead, the colon should be divided at that point and brought out as a diverting colostomy. The distal colon should not be resected but closed off and tacked close to the ostomy site. Before reversal, both proximal and distal colon should be assessed with contrast studies to determine how much colon should be resected. Thus, definitive resection and anastomosis may better be judged and performed in a delayed setting when sepsis, edema and inflammatory changes have settled. Given these multiple factors with regard to the health of the gut, as described in the 2014 ASCRS guidelines, the decisions on the surgical procedure should be made on a case-by-case basis [2].

In our patient, the colon was severed at the point of the perforation and exteriorized. This allowed time for the gut inflammatory changes to settle. A contrast study performed four weeks later revealed that the majority of the sigmoid colon was in fact healthy although at the time of perforation, the sigmoid and mesentery were both highly edematous. If a Hartmann’s operation was done, this patient would have been subjected to unnecessary removal of healthy colon and condemned to a colostomy with a very low possibility of reversal.

We recommend that in cases of perforated diverticular disease, a primary repair of the defect should be performed if the colon is healthy at the time of operation. If there is a small portion of the diseased colon, a resection with primary anastomosis can be considered. However, if the sigmoid appears unhealthy at the time of operation, a diverting colostomy should be done with transection and colostomy at the site of perforation; this will allow the sepsis and inflammatory process to settle before the definitive resection that can be planned using contrast studies of the proximal and distal colon.

## Conclusions

When the colon appears diseased on the initial emergency operation for PDD, if given the time to rest, the sigmoid is revealed to be normal on contrast imaging done to plan reversal of the stoma. Thus, Hartmann’s operation for diverticular disease should be abandoned to avoid unnecessary resection of the healthy gut and to prevent patients from being condemned to a stoma that will likely never be reversed while also reducing morbidity and mortality rates. Even when primary resection and anastomosis is considered, to avoid resecting the healthy colon that appears diseased at the time of operation, diverting colostomy should be performed to allow the gut to recover before definitive management is planned.
